# Genomic and Proteomic Characterization of Bacteriocin-Producing *Leuconostoc mesenteroides* Strains Isolated from Raw Camel Milk in Two Southwest Algerian Arid Zones

**DOI:** 10.1155/2014/853238

**Published:** 2014-04-07

**Authors:** Zineb Benmechernene, Inmaculada Fernández-No, Marcos Quintela-Baluja, Karola Böhme, Mebrouk Kihal, Pilar Calo-Mata, Jorge Barros-Velázquez

**Affiliations:** ^1^Laboratory of Applied Microbiology, Department of Biology, Faculty of Sciences, Oran University, B.P. 16, 31100 Es-Senia, Oran, Algeria; ^2^Department of Analytical Chemistry, Nutrition and Food Science, School of Veterinary Sciences/College of Biotechnology, University of Santiago de Compostela, Rúa Carballo Calero s/n, Campus Lugo, 27002 Lugo, Spain

## Abstract

Information on the microbiology of camel milk is very limited. In this work, the genetic characterization and proteomic identification of 13 putative producing bacteriocin *Leuconostoc* strains exhibiting antilisterial activity and isolated from camel milk were performed. DNA sequencing of the 13 selected strains revealed high homology among the 16S rRNA genes for all strains. In addition, 99% homology with *Leuconostoc mesenteroides* was observed when these sequences were analysed by the BLAST tool against other sequences from reference strains deposited in the Genbank. Furthermore, the isolates were characterized by matrix-assisted laser desorption/ionization time of flight mass spectrometry (MALDITOF MS) which allowed for the identification of 2 mass peaks 6242 m/z and 5118 m/z that resulted to be specific to the species *L. mesenteroides*. Remarkably, the phyloproteomic tree provided more intraspecific information of *L. mesenteroides* than phylogenetic analysis. Accordingly, phyloproteomic analysis grouped *L. mesenteroides* strains into different subbranches, while all *L. mesenteroides* isolates were grouped in the same branch according to phylogenetic analysis. This study represents, to our knowledge, the first report on the use of MALDI-TOF MS on the identification of LAB isolated from camel milk.

## 1. Introduction

Increasing consumer demand for natural, healthy, and convenient foods has resulted in a new generation of minimally processed foods that focus on biopreservation, refrigeration, and packaging as hurdle strategies to extend the shelf-life of these products. The use of natural antimicrobial metabolites from lactic acid bacteria (LAB) has been determined to be one of the most promising strategies in minimal processing. LAB are food-grade organisms that may be used as an alternative to chemical preservatives in biopreservation strategies due to their ability to produce several antimicrobial compounds, including organic acids, hydrogen peroxide, and bacteriocins [1].* Leuconostoc *spp. and other LAB strains isolated from meat or dairy products produce bacteriocins that are active against the major food pathogen* Listeria monocytogenes* [2–6]. Although this activity was first observed in the 1950s, extensive studies on bacteriocins produced by* Leuconostoc *spp. have only been conducted in the last 25 years. The importance of* Leuconostoc* strains in the dairy industry is widely recognized; however, knowledge of their physiology and genetics is less developed than that of* Lactococcus* [7].

Traditional dairy products such as LAB represent a reservoir of phenotypic and genetic microbial diversity, which may have biotechnological applications [8–10]. To date, raw camel's milk has been underinvestigated as a potential source of food-grade LAB and has not generated a large industrial interest. One of the main reasons for the underinvestigation of raw camel milk is that the world production of camel milk for human consumption was recently estimated to only be 1.3 million tons/year [11]. Algeria produces only 8.100 tons/year of camel milk, but other countries such as Saudi Arabia (90.000 tons/year) and Sudan (82.250 tons/year) are strong producers. The majority of scientific studies on camels have been mainly focused on their anatomic characteristics and physiological adaptation to adverse climates. Consequently, information regarding camel milk is very limited. Previous studies on the molecular characterization of LAB isolated from fermented camel milk have been reported in the Xinjiang region of China [12], on the isolation of* Lactococcus lactis* from Algerian camel milk [13] and on the isolation of* L. mesenteroides* from fermented camel milk, “Raib” [14]. However,* L. mesenteroides* strains isolated from raw Algerian camel milk have not been characterized.

In the present study, raw camel milk was chosen because of its beneficial effects on human health [15], such as its antibacterial activity [16], antiviral activity [17] (Redwan and Tabll 2007), anti-inflammatory activity [18], anticancer activity [19], and antiallergic activity [20]. Additionally, camel milk is known for its extended shelf-life, which allows for storage and safe consumption after several days in the absence of refrigeration [21].

*Leuconostoc* and other LAB traditionally have been characterized phenotypically. However, new molecular techniques have been proposed for* leuconostocs* and other LAB to avoid the limitations of phenotypic characterization to achieve reliable and consistent identification. Therefore, 16S rRNA-based amplification and sequencing methods have been reported for the characterization of* leuconostocs* by Lee et al. [22], Schönhuber et al. [23], Pérez et al. [24], Randazzo et al. [25], Dal Bello et al. [26], Ennahar et al. [27], Kim et al. [28], and Reeson et al. [29]. More recently, proteomic tools such as matrix-mssisted laser desorption/ionization time of flight mass spectrometry (MALDI-TOF MS) have also been proposed for bacterial identification purposes. These proteomic tools offer high throughput (95%–97.4% correct identifications) [30, 31] and produce unprecedented levels of discrimination among bacterial species and strains [32–35]. Therefore, the objective of this study was to isolate and identify* L. mesenteroides* strains exhibiting antibacterial activity from Algerian raw camel milk and use MALDI-TOF MS to determine protein biomarkers useful for the specific identification and classification of* L. mesenteroides*.

## 2. Material and Methods

### 2.1. Raw Camel Milk Sampling

The 13* Leuconostoc *strains considered in this work were isolated from four different camel milk samples, which were collected at two different sampling times (2009 and 2011) from two different Algerian arid zones situated in the southwest of Algeria. The first zone, called Nâama, is located at 432 km away from the capital Algiers; the second zone, Abadla, close to the city of Béchar, is situated at 1150 km away from the capital. The first two samples were collected from two camels (*Camelus dromados*) in Nâama which were in the range of 10–15 years old and coloured in grey and black, respectively. Both camels had the same lactation period, which was in March 2011. The diet of these camels was based on natural Saharan plants, called drinn (*Aristida pungens*). Samples from Béchar were collected at Abadla in 2009 and 2011 from brown camels aged less than 10 years that have a daily production of 6 to 9 milk liters. In all cases, sampling was performed under aseptic conditions by washing the teats with warm water containing 2% bleach and collecting milk in sterile glass bottles after hand washing with diluted alcohol. Samples were then transported by airplane to the laboratory in a cool box and stored at 4 ± 1°C until analysis. The samples were analyzed within 12 to 30 hrs after collection.

### 2.2. Bacterial Strains and Culture Conditions

The bacteriocin-producing* leuconostocs* considered in this work were isolated from raw camel milk as described above. All strains were stored at −80°C in reconstituted skimmed milk containing 30% (w/v) glycerol. All strains were cultured in MRS broth (Liofilchem, Teramo, Italy) at 30°C for 24 h and were then seeded onto MRS agar (Liofilchem) to obtain single colonies. Ten wild-type and reference* leuconostoc* strains used in this study are shown in Table 1. Thus, five reference strains were considered: three from the Spanish Type Culture Collection and two from the Ghent University Type Culture Collection (Table 1).

### 2.3. Phenotypic Characterization of Isolates

Fifteen strains were selected and subjected to the following physiological tests on the basis of the following phenotypic and morphological criteria: CO_2_ production, growth at different temperatures (4°C, 15°C, 30°C, 37°C, and 45°C), growth at different pH (4.8 and 6.8), and growth at different NaCl concentrations (3% and 6.5%). Additionally, all strains were subjected to the following biochemical tests in order to differentiate between* leuconostocs* and* lactobacilli*: dextran production on MSE medium [36], arginine hydrolysis on M16BCP medium (Oxoid Ltd., London, UK), and citric acid degradation on Kempler and McKay solid medium. Carbohydrate fermentation was performed on MRS supplemented with bromocresol purple as a pH indicator by using the following sugars to differentiate between the following subspecies of* leuconostocs*: arabinose, maltose, rhamnose, esculine, mannitol, sorbitol, galactose, lactose, fructose, glucose, sucrose, and xylose. All strains considered in this study were phenotypically identified as belonging to the* Leuconostoc* genus based on the following criteria: ovoid shape, Gram-positive, catalase negative, vancomycin-resistant, production of gas from glucose, no arginine hydrolysis, and by their fermentation profiles.

### 2.4. Inhibition Assays of Indicator Microorganisms

Preliminarily, all strains were tested for their ability to produce antimicrobial substances by the direct method described by Fleming et al. [37]. Inhibitory activity was investigated on the following indicator bacteria:* Lactobacillus plantarum, Lactococcus *sp. (LMA, Oran, Algeria),* Escherichia coli*: 25922,* Staphylococcus aureus*: 43300 (Centre Hospitalier Universitaire, C.H.U Oran, Algeria),* Listeria innocua* (ATCC 33090), and* Listeria ivanovii* (ATCC 19119). Aliquots of 18 h cultures of each* Leuconostoc* strain were spotted on MRS agar using multipoint inoculators and were incubated at 30°C for 24 h [38]. Following incubation, a semisolid Mueller Hinton (Oxoid) medium containing 100 *μ*L of 10^7^ CFU mL^−1^ of indicator culture was poured as an overlay. All plates were then incubated at 37°C for 24 h and examined for the formation of inhibition zones. Inhibition was considered positive when the width of the clear inhibition halos was ≥ 0.5 cm.

### 2.5. Genetic Identification of* Leuconostoc* Strains, Phylogenetic Analysis, and Clustering

Total genomic DNA was extracted and purified using the DNeasy Tissue Mini Kit (Qiagen, Valencia, CA) [39]. Briefly, this method utilized the purification of DNA using microcolumns and its final recovery using a commercially prepared elution buffer. A fragment of the 16S rRNA gene was amplified by PCR using the universal primer pair p8FPL (forward: 5′-AGTTTGATCCTGGCTCAG-3′) and p806R (eeverse: 5′-GGACTACCAGGGTATCTAAT-3′) [40]. All PCR assays were conducted on a “My Cycler” Thermal Cycler (BioRad Laboratories, Hercules, USA). The assays comprised 100 ng of template DNA, 25 *μ*L of a master mix (BioMix, Bioline, London, UK) (this included the reaction buffer, dNTPs, and magnesium chloride), Taq DNA polymerase, 25 pmol of each oligonucleotide primer, and double-distilled water to achieve a final volume of 50 *μ*L. Amplification conditions were as follows: denaturing at 94°C for 7 min, 35 cycles of denaturation (94°C for 60 sec), annealing (55°C for 60 sec), extension (72°C for 60 sec), and a final extension at 72°C for 15 min. The PCR was performed as described by Böhme et al. [41].

Prior to sequencing, PCR products were purified with the “EXOSAP-IT” Kit (GE Healthcare, Uppsala, Sweden). Direct sequencing was performed with the “Big Dye Terminator v 3.1” Cycle Sequencing Kit (Applied Biosystems, Foster City, CA). The same primers used for PCR were also used for sequencing both strands of the PCR products. The sequencing reactions were analysed in an automatic sequencing system (ABI 3730XL DNA-Analyzer, Applied Biosystems) with the POP-7 system. All 16 rRNA gene sequences were analysed with Chromas software (Griffith University, Queensland, Australia) and aligned using Clustal X software [42]. Following alignment, these sequences were identified by searching for sequence homology among published reference sequences using the web BLAST tool (National Center for Biotechnology Information (NCBI), http://blast.ncbi.nlm.nih.gov/) [43]. Homologies higher than 99% with respect to a strain type were considered good identifications.

Phylogenetic and molecular evolutionary analyses were conducted with MEGA 5.0 software [44]. Phylogenetic clustering and construction of a phylogenetic-based tree were performed using the neighbour-joining method [42] by using the “Bootstrap method” as a test of phylogeny and the “Kimura 2-parameter model” to compute the evolutionary distances [45, 46]. The bootstrap consensus tree inferred from 1000 replicates was taken to represent the evolutionary history of the taxa analysed [47]. Meanwhile, estimates of evolutionary divergence and diversity values for 16S rRNA gene sequences were conducted with the MEGA 5.0 software using the “Maximum composite likelihood model” [46–48].

### 2.6. MALDI-TOF MS and Phyloproteomic Analysis of* Leuconostoc* Isolates

The 13* Leuconostoc* strains whose 16S rRNA had been sequenced were grown on MRS agar plates for 24 h. Then, a 1 *μ*L loop of each bacterial culture was harvested and placed in 100 *μ*L of a solution consisting of 50% acetonitrile (ACN) (Merck, Darmstadt, Germany) and 1% aqueous trifluoroacetic acid (TFA) (Acros Organics, Morris Plains, NJ). The bacterial pellet was vortexed at least two times until the pellet was completely resuspended. Complete homogenization of the mixture was required to obtain good spectral profiles for* leuconostocs*. After centrifugation at 8000 rpm for 10 min, the supernatants were transferred to new tubes and stored at −20°C. A 1 *μ*L aliquot of each sample solution was mixed with 10 *μ*L of a matrix solution consisting of 10 mg *α*-cyano-4-hydroxycinnamic acid (*α*-CHCA) in 1 mL of 50% ACN and 2.5% aqueous TFA. From this final solution of sample and matrix, a 1 *μ*L aliquot was manually deposited onto a stainless steel plate and allowed to dry at room temperature.

Mass spectra were obtained using a Voyager “DE STR MALDI-TOF” Mass Spectrometer (Applied Biosystems, Foster City, CA) operating in a linear mode and extracting positive ions with an accelerating voltage of 25,000 V and delay time of 350 ns. The grid voltage and guide wire were set to 95% and 0.05%, respectively. Each spectrum was the accumulated sum of at least 1000 laser shots, which were obtained from 10 different regions and manually selected from the same sample spot in a range of 1500–15000 Da. For every strain, two extractions were performed and both extracts were measured in duplicate totalling of four spectra for each bacterial strain. The mass spectra were externally calibrated using a mixture of 1 pmol/*μ*L oxidized insulin B-chain and 1 pmol/*μ*L bovine insulin (Sigma-Aldrich, St. Louis, MO) that were analysed with Data Explorer software (version 4.0) (Applied Biosystems) for baseline correction and noise filtering.

After obtaining four spectral profiles for each bacterial strain, mass spectra were analysed with Data Explorer software (version 4.0), baseline corrected, and noise filtered. Data lists containing m/z values were extracted from mass spectral data, including signals with relative intensities higher than 2%. The obtained peak mass lists were analysed and compared using peaks in the mass range of 2000–10000 Da because of the reproducibility of the spectral profile in that mass range.

Mass lists were further processed with the free web-based application SPECLUST, which is available at “http://bioinfo.thep.lu.se/speclust.html” [49]. The “peaks in common” option in this web interface calculates the mass difference between four peaks taken from different peak lists and determines if two peaks are identical after taking into account measurement uncertainty (*σ*) and peak match score (*s*). The peak match score represents the probability that two peaks with measured masses *m* and *m*′ have a mass difference equal or larger than |*m* − *m*′| given that the mass difference is only due to measurement errors. Because each bacterial strain was cultured in duplicate and each culture was analysed in duplicate, this tool was used to examine the four spectra from each sample. The representative common peaks present in all four spectra were extracted by this web application with a peak match score greater than 0.7 (which corresponds to a measurement error of ±5 Da) to obtain species-specific and genus-specific biomarkers. A peak was considered to be common to four spectra if the peak match score was larger than 0.7, which corresponded to a range in peak match score of 10 Da. According to these specifications, specific mass lists were generated for every bacterial strain (including 5–35 peak masses), which represented reproducible bacterial fingerprints.

Mass lists of all* leuconostocs* were clustered by using the “clustering” option that is also available in the web interface SPECLUST. The agglomerative clustering method created one cluster for every peak list and calculated distances between clusters. The two clusters with the smallest maximum pairwise distance (complete linkage) were then merged into a new cluster and the distances were calculated again by adding all individual similarity scores for every pair of two peak lists. This process was repeated until one single cluster remained. All individual similarity scores of each pair of the two peak lists were added up to calculate the distances between the two peak lists. The width in the peak match score was set to 10 Da. Resulting distances varied between “1” for completely different set of peak masses and “0” for perfect matches.

Finally, phyloproteomic clustering was confirmed through the analysis of mass lists using Statgraphics Plus software (version 5.1). The mass list table was transformed into a binary table, which was followed by clustering by using centroid and group average analytical methods and by using block population distance metric and cluster variable options.

## 3. Results

### 3.1. Phenotypic Characterization of* Leuconostoc* Isolates from Raw Camel Milk

Macroscopic observation of bacterial colonies led to the selection of 15 observably different 0.5–1.5-mm-wide white small colonies that had a lenticular shape on MRS agar supplemented with vancomycin. All of the colonies exhibited a glutinous transparent aspect on MSE agar. All 15 isolates were Gram-positive and catalase negative, exhibited ovoid shape, and were associated with short pairs and/or chains. Additionally, all isolates were citrate positive, were able to produce CO_2_ from glucose, were able to produce dextran from sucrose, and were unable to hydrolyse arginine. Furthermore, all isolates were able to grow at 15°C, 30°C, and 37°C but were unable to grow at 4°C and 45°C. All isolates were resistant to 3% NaCl and to pH 6.8. None of the isolates were able to grow on 6.5% NaCl at pH 4.8. Fermentation profiling showed that the 13 strains that exhibited antilisterial activity were able to ferment glucose and lactose, but these strains exhibited some differences in their ability to ferment other sugars (Table 2).

### 3.2. Antimicrobial Activity of* Leuconostoc* Isolates

Thirteen of the 15 isolates exhibited inhibitory activity against other LAB such as* Lactobacillus *spp. and* Lactococcus *spp. and against several pathogenic bacteria, such as* E. coli*: 25922*, S. aureus*: 43300, * L. innocua* (ATCC 33090), and* L. ivanovii* (ATCC 19119). The inhibition zones were measured and their diameters are compiled in Table 3. The results of inhibition indicated that the inhibition intensity and range varied depending on the* leuconostoc* species assayed.

**Figure 1:**
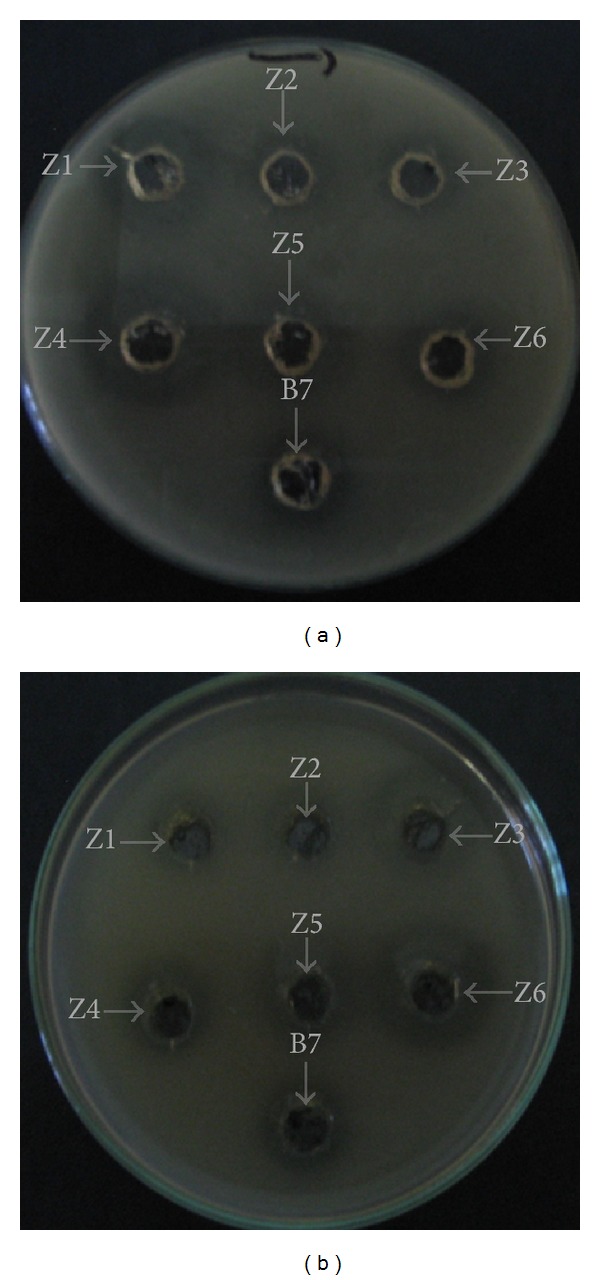
Antibacterial activity after eliminating lactic acid with buffered medium. (a)* Leuconostoc mesenteroides* effect on* Listeria innocua.* (b)* Leuconostoc mesenteroides* effect on* Listeria ivanovii*. Z1–Z6:* L. mesenteroides* isolated from camel milk sample 1 and B7:* L. mesenteroides* isolated from camel milk sample 2.

Furthermore, to investigate whether the cause of the inhibition was due to protein, buffered supernatants adjusted to pH 6.8 were treated with chymotrypsin, which lead to the disappearance of inhibition zones. This result indicated that inhibition was caused by a proteinaceous compound (Figure 1). However, inhibition remained after heating the bacterial supernatants to a temperature of 100°C (data not shown), which indicated that the causative inhibitory agent is heat resistant. These results agreed with previous results reported by Lachance [50] and Labioui et al. [51].

### 3.3. Phylogenetic Analysis of* Leuconostoc* Isolates

**Figure 2:**
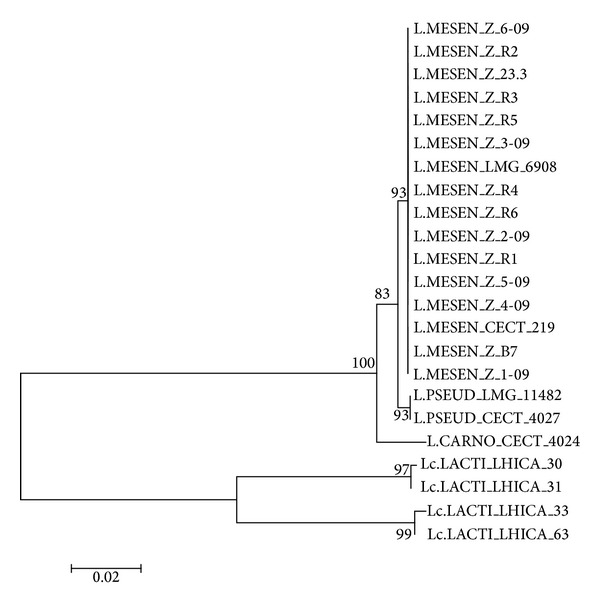
Phylogenetic tree of* Leuconostoc* spp. This tree was generated for* Leuconostoc* spp. isolated from camel milk and other reference strains based on 16S rRNA nucleotide sequences using the neighbour-joining method. Numbers above and below branches indicate bootstrap values from the neighbour-joining analysis.

DNA sequencing of the 13 selected isolates revealed high homology among their 16S rRNA nucleotide sequences. In addition, sequence analysis by the BLAST tool against other sequences from reference strains deposited in the GenBank revealed a 99% homology with* L. mesenteroides*. A phylogenetic tree was constructed by considering other* Leuconostoc* and* Lactococcus *reference and collection strains. These results are presented in Figure 2. Thus, phylogenetic analysis indicated that all strains isolated from raw camel milk were grouped in a common branch with reference strains* L. mesenteroides* (CECT 219) and* L. mesenteroides* (LMG 6908). This confirmed the identity of such strains as* L. mesenteroides*. However,* Leuconostoc pseudomesenteroides* (LMG 11482) and* L. pseudomesenteroides* (CECT 4027) were grouped together but were not in the same cluster as the* L. mesenteroides* strains. Additionally,* Leuconostoc carnosum* (CECT 4024) clustered in another branch separate from the other two.* Lactococcus* strains clustered into two distinct subclusters corresponding to (i)* Lactococcus lactis* subsp.* cremoris*, which included the* L. lactis* (LHICA 63) and* L. lactis* (LHICA 33) strains, and (ii)* L. lactis* subsp.* lactis*, which included the* L. lactis* (LHICA 30) and* L. lactis* (LHICA 31) strains. These two subclusters were separated by a short distance due to their high genetic similarity as compared to* L. carnosum*,* L. mesenteroides,* and* L. pseudomesenteroides*.

### 3.4. MALDI-TOF MS Fingerprinting of* Leuconostoc* Isolates

Identification of* leuconostocs* was also performed by MALDI-TOF MS. Four spectra were obtained for each strain. The search for common peak masses in the spectra was performed using the SPECLUST application. Arithmetic means were calculated for m/z values and the standard deviation was calculated to be ±5 Da. The mass lists include 68 peak masses that were generated for 19* Leuconostoc* strains with four* Lactococcus* strains classified as an outgroup. While 46 peaks were only observed in* Leuconostoc* strains, 20 peaks were specific to the* Lactococcus* genus and only two peaks were shared by both genera.

**Figure 3:**
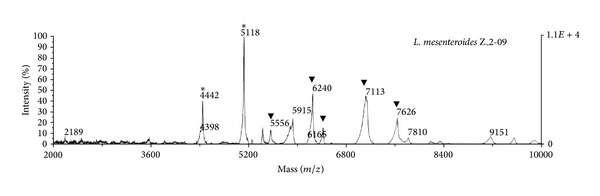
Characteristic mass peaks of* L. mesenteroides* strain Z_2-09 isolated from camel milk. Specific genus peaks marked as (∗) are present in other* Leuconostoc* strains and peaks marked as (*▼*) are absent in the* Lactococcus* genus.

**Figure 4:**
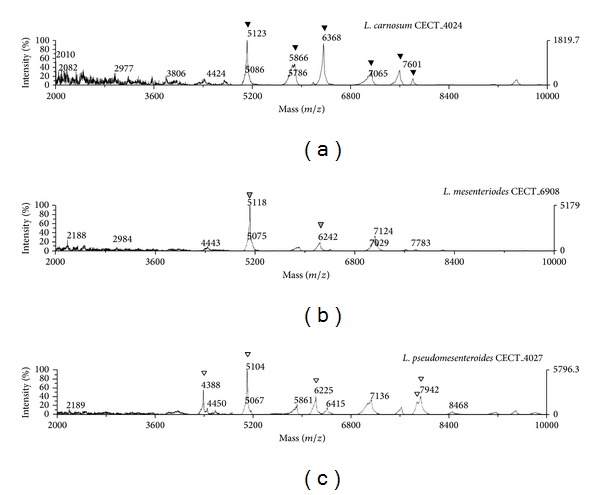
Spectra corresponding to the species* L. carnosum*,* L. mesenteroides,* and* L. pseudomesenteroides*. Species-specific peaks are marked in each case by (*▽*).

Remarkably, the spectral profile (fingerprinting) revealed different results for* Lactococcus* and* Leuconostoc* genera. The highest intensity peak in* Lactococcus* appeared at m/z 3865 Da, while the highest intensity peak in* Leuconostoc* appeared at m/z 5118 Da. Significant differences in the mass peak lists between these two genera were observed (Figure 3). The spectral profiles for the different* Leuconostoc* spp. shared a great number of peaks, but there were some differences in the presence/absence of peaks (Figure 4). Thus, the peak at m/z 6242 Da was present in both* L. mesenteroides *and* L. pseudomesenteroides* but was shifted at 6368 Da in* L. carnosum.* Of the 46 peaks present in the* Leuconostoc* genus, 10 were present in more than 50% of the samples analysed. It should be noted that the peak at m/z 4442 Da was present in all* Leuconostoc* spp. with the exception of strain R1, which is probably due to slight differences in the protein amino acid sequence [52]. Therefore, the peaks at m/z 4442 Da and m/z 5118 Da are specific for the* Leuconostoc *genus (Table 4). A phyloproteomic tree was constructed from the peak mass list (Figure 3) by using the SPECLUST program to differentiate between the* Leuconostoc* spp. isolated from raw camel milk. Thus, two main clusters were observed in the dendrogram: one cluster corresponded to the* Lactococcus* genus, which was considered an outgroup, while the other cluster included all three* L. carnosum*,* L. pseudomesenteroides,* and* L. mesenteroides* species. Remarkably, the phyloproteomic tree provided more intraspecific information for* L. mesenteroides* than 16S rRNA-based phylogenetic analysis. The phyloproteomic analysis allowed the* L. mesenteroides* strains to be grouped into different subbranches, while all* L. mesenteroides* isolates were grouped in the same branch according to phylogenetic analysis.

## 4. Discussion

Camel milk is an important food in arid and semiarid regions where it covers most qualitative and quantitative nutritional needs. While many studies have investigated the microbiology of cow, sheep, and goat's milk, only a few studies have focused on the microbiology of camel milk. Other authors have reported the effectiveness of protective proteins from camel milk against bacteria, such as* L. lactis* subsp.* cremoris*,* E. coli*,* S. aureus*,* Salmonella typhimurium,* and* rotavirus* [16]. The inhibition of pathogenic bacteria by protective proteins such as lysozyme, lactoperoxidase, or lactoferrin naturally present in camel milk has also been previously described by Barbour et al. [53].

Remarkably, only a few studies have addressed the genetic identification of LAB isolated from camel milk and these studies analysed other regions of the world [54–56]. Therefore, to the best of our knowledge, no genetic information regarding* Leuconostoc* spp. isolated from raw camel milk in northern Africa has been previously reported. Additionally, the use of MALDI-TOF MS for the characterization of LAB isolated from camel milk has never been performed before and only one study regarding the proteomic identification of* Leuconostoc* from other food sources has been performed by de Bruyne et al. [57]. The present study focused on the characterization and proteomic identification of* Leuconostoc *spp. from Algerian raw camel milk.* Leuconostoc *spp. act as starter cultures and also exert beneficial effects on the microbiological stability and production of aroma compounds in various food products. More importantly,* Leuconostoc *spp. play a crucial role in food biopreservation through the production of bacteriocins with different inhibition spectra (they are especially effective as antilisterial agents) [58].

The* L. mesenteroides *isolated in this work exhibited significant inhibition against indicator strains (Table 3). This inhibition was not caused by the production of organic acids, hydrogen peroxide, or lysogenic phages as the molecules responsible for inhibition were sensitive to protease treatment. Phenotypic, genotypic, and proteomic analysis revealed that the 13* L. mesenteroides* isolates from raw camel milk were identical. Additionally, according to 16S rRNA gene sequencing, the 13 strains exhibited high similarity among themselves and with respect to other sequences from reference strains deposited in the GenBank. Moreover, phylogenetic analysis revealed that all 13 isolates clustered in the same branch, which confirms their clonal homogeneity.

Finally, this study represents the first report on the application of MALDI TOF MS analysis for the faster and more reliable identification of* L. mesenteroides* strains isolated from Algerian raw camel milk based on their low-molecular-weight protein profile. The spectra were generated in quadruplicate to ensure the reproducibility of these results. The small differences in the spectra of individual strains may be caused by bacterial response to stress and environmental changes, including storage and handling. Small spectral differences are observed in the majority of cases and can cause slight differences in the intensity of some peaks [59]; however, relevant peaks are rarely affected. This was also observed in our study (data not shown).

**Figure 5:**
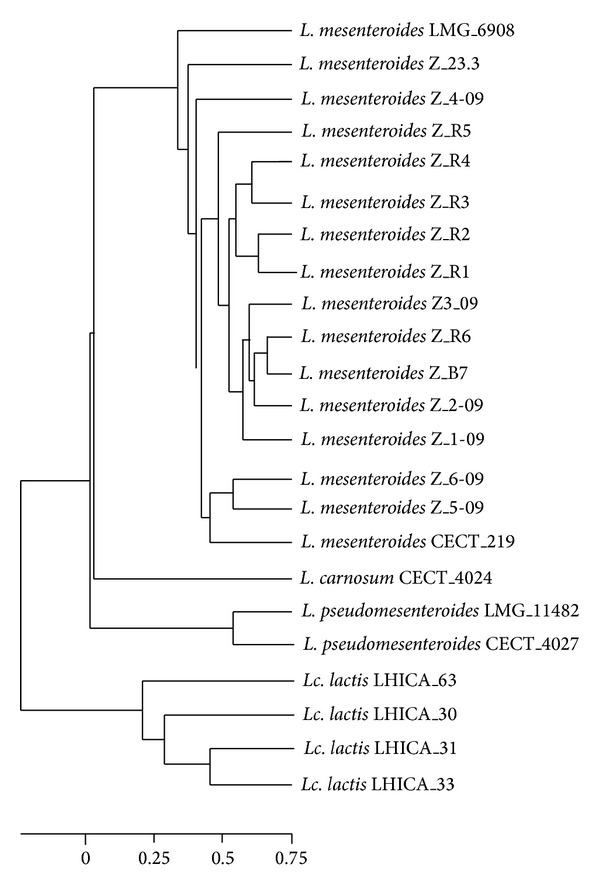
Phyloproteomic tree of* Leuconostoc* spp. based on the protein profile obtained by MALDI-TOF MS.

The mass spectrometry profiles obtained for each* Leuconostoc* sp. allowed for the generation of species-specific peak mass lists (Table 4) for* L. mesenteroides*, * L*.* pseudomesenteroides,* and* L. carnosum* (Table 1). Remarkably, these results allowed for the identification of peak masses specific to* L. mesenteroides* that could serve as biomarker peaks in future analyses. Moreover, the phyloproteomic tree proposed in this study for* Leuconostoc* spp. isolated from raw camel milk provided more intraspecific information than a 16S rRNA-based phylogenetic analysis of* L. mesenteroides*. Therefore, phyloproteomic analysis allowed for the grouping of* L. mesenteroides* strains into different subgroups (Figure 5), while the phylogenetic proximity of these strains did not allow for such differentiation [57].

In summary, this study has provided the first genetic characterization of bioactive* Leuconostoc *spp. isolated from Algerian raw camel milk. Additionally, the application of MALDI TOF peptide mass fingerprinting was successfully applied to this bacterial group and proved to be a simple, quick, and inexpensive complementary method for bacterial identification at the species level.

## Figures and Tables

**Table 1:** Reference strains considered in the phylogenetic and proteomic studies.

*Leuconostoc* spp.			
Species	Source	Origin	Code
*Leuconostoc pseudomesenteroides *	CECT 4027	Juice	L.PSEUD_CECT_4027
*Leuconostoc mesenteroides *	CECT 219	Fermented olives	L.MESEN_CECT_219
*Leuconostoc carnosum *	CECT 4024	Beef meat	L.CARNO_CECT_4024
*Leuconostoc mesenteroides *	LMG 6908	ND	L.MESEN_LMG_6908
*Leuconostoc pseudomesenteroides *	LMG 11482	ND	L.PSEUD_LMG_11482
*Lactococcus lactis *	LHICA	Cow milk	Lc.LACTI_LHICA_30
*Lactococcus lactis *	LHICA	Cow milk	Lc.LACTI_LHICA_31
*Lactococcus lactis *	LHICA	Cow milk	Lc.LACTI_LHICA_33
*Lactococcus lactis *	LHICA	Cow milk	Lc.LACTI_LHICA_63
*Leuconostoc 23.3 *	LHICA	ND	L.MESEN_LHICA_Z_23.3

CECT: Spanish Type Culture Collection; LMG: Ghent University Type Culture Collection; LHICA: University of Santiago LHICA Bacterial Collection; and ND: not determined.

**Table 2:** Fermentation profiling of *Leuconostoc* strains isolated from camel milk.

Strains	Ara	Mal	Rha	Esc	Man	Sorb	Gal	Lac	Fru	Glu	Sac	Xyl
Z_1-09	+	−	+/−	+/−	−	−	+	+	+	+	+	+
Z_2-09	+	+	−	+	+	−	+	+	+	+	+	+
Z_3-09	+	+	−	−	+	−	+	+	+	+	+	+
Z_4-09	+	+	−	+	+	+/−	+	+	+	+	+	+
Z_5-09	+	+	−	+	+	+	+	+	+	+	+	+
Z_6-09	+	+/−	−	+/−	−	+/−	+	+	+	+	+	+
zB7	−	+	−	−	−	−	+	+	+	+	+	+
Z_R1	+	+	NI	−	−	−	+	+	+	+	+	+
Z_R2	+	+/−	NI	−	−	−	+/−	+	+	+/−	+/−	+
Z_R3	+	+/−	NI	+/−	+/−	−	+/−	+	+	+	+/−	+/−
Z_R4	+	+/−	NI	−	−	−	+	+	+	+/−	−	+
Z_R5	−	+	NI	−	−	−	+	+	+	+	+	+
Z_R6	+	+/−	NI	−	+/−	−	+	+	+	+	+	+

NI: not identified.

**Table 3:** Diameters of the inhibition zones of *Leuconostoc* strains (Z1_09 to B7) isolated from camel milk on indicator strains.

Strains	Z_1-09	Z_2-09	Z_3-09	Z_4-09	Z_5-09	Z_6-09	R1	R2	R3	R4	R5	R6	B7
*Lactobacillus plantarum *	7	8	11	8	9	10	NI	NI	NI	NI	NI	NI	9
*Lactococcus* sp.	8	6	8	8	7	7	20	17	20	20	20	20	5
*Escherichia coli *	8	9	7	10	10	8	15	12	16	20	18	17	7
*Staphylococcus aureus *	8	8	7	9	11	8	18	20	25	27	17	20	8
*Listeria innocua *	8	6	9	9	8	10	10	11	10	10	8	10	9
*Listeria ivanovii *	8	9	7	9	11	10	10	7	6	11	—	—	8

**Table 4:** List of species-specific peak masses of *L. carnosum*, *L. pseudomesenteroides,* and *L.mesenteroides*.

Microbial species		
*L. carnosum *	*L. pseudomesenteroides *	*L. mesenteroides *
4424	4388	6242
5123	5104	5118
5866	6225	
6368	7942	
7065		
7601		
